# JOURNAL OF GERONTOLOGY SOCIAL SCIENCES: FEATURED 2022 EDITOR’S CHOICE ARTICLES

**DOI:** 10.1093/geroni/igad104.0439

**Published:** 2023-12-21

**Authors:** Jessica Kelley

## Abstract

Social science inquiry on age, aging, and the life course spans many topics and methodologies. This symposium highlights papers that were selected as Editor’s Choice articles in the Journal of Gerontology Social Sciences in 2022. These papers highlight methodological innovations, important advancements in our state of knowledge in an area, or emerging issues in the study of aging and older adults. Moen et al. trace the divergence in later-adulthood work trajectories at the intersection of race, gender, and class. Hamler et al. discuss the impact of skin tone on mental health among older Black Americans. Waselmann et al. present an innovation of counting number of days one attended school and whether one lived in the Jim Crow South to help explain Black-White disparities in later-life cognitive function. Falzarano et al. explore cultural differences in orientation toward familialism and its impact on caregiver outcomes. Zimmer et al. examine the linkage between war exposure and later-life frailty among Vietnamese older adults.

